# Burden of salmonellosis, campylobacteriosis and listeriosis: a time series analysis, Belgium, 2012 to 2020

**DOI:** 10.2807/1560-7917.ES.2017.22.38.30615

**Published:** 2017-09-21

**Authors:** Charline Maertens de Noordhout, Brecht Devleesschauwer, Juanita A Haagsma, Arie H Havelaar, Sophie Bertrand, Olivier Vandenberg, Sophie Quoilin, Patrick T Brandt, Niko Speybroeck

**Affiliations:** 1 Institute of Health and Society (IRSS), Université catholique de Louvain, Brussels, Belgium; 2 Department of Public Health and Surveillance, Scientific Institute of Public Health (WIV-ISP), Brussels, Belgium; 3 Department of Public Health, Erasmus MC, Rotterdam, the Netherlands; 4 University of Florida, Gainesville, Florida, United States; 5 Utrecht University, Utrecht, the Netherlands; 6 Université libre de Bruxelles, Brussels, Belgium; 7 University of Texas, Dallas, Richardson, Texas, United States

**Keywords:** Salmonellosis, campylobacteriosis, listeriosis, Belgium, burden, time series, prediction, DALYs

## Abstract

Salmonellosis, campylobacteriosis and listeriosis are food-borne diseases. We estimated and forecasted the number of cases of these three diseases in Belgium from 2012 to 2020, and calculated the corresponding number of disability-adjusted life years (DALYs). The salmonellosis time series was fitted with a Bai and Perron two-breakpoint model, while a dynamic linear model was used for campylobacteriosis and a Poisson autoregressive model for listeriosis. The average monthly number of cases of salmonellosis was 264 (standard deviation (SD): 86) in 2012 and predicted to be 212 (SD: 87) in 2020; campylobacteriosis case numbers were 633 (SD: 81) and 1,081 (SD: 311); listeriosis case numbers were 5 (SD: 2) in 2012 and 6 (SD: 3) in 2014. After applying correction factors, the estimated DALYs for salmonellosis were 102 (95% uncertainty interval (UI): 8–376) in 2012 and predicted to be 82 (95% UI: 6–310) in 2020; campylobacteriosis DALYs were 1,019 (95% UI: 137–3,181) and 1,736 (95% UI: 178–5,874); listeriosis DALYs were 208 (95% UI: 192–226) in 2012 and 252 (95% UI: 200–307) in 2014. New actions are needed to reduce the risk of food-borne infection with *Campylobacter* spp. because campylobacteriosis incidence may almost double through 2020.

## Introduction

Salmonellosis in humans is caused by non-typhoidal *Salmonella enterica,* bacteria originating in animal reservoirs that can spread to humans through contaminated foods, such as eggs as well as raw meat from pigs and chickens, and through non-food pathways, such as direct contact with infected animals or humans [1,2]. The most common symptoms of human salmonellosis include fever, diarrhoea and abdominal cramps, but if bacteria invade the bloodstream, they can cause septicaemia and even death. Irritable bowel syndrome (IBS), inflammatory bowel disease (IBD) and reactive arthritis are possible consequences of salmonellosis [2]. For 2010, the World Health Organization (WHO) estimated that non-typhoidal salmonellosis caused three disability-adjusted life years (DALYs) per 100,000 population (95% uncertainty interval (UI): 2–5) in the WHO European Region [3,4].

Campylobacteriosis is mainly caused in humans by the Gram-negative bacteria *Campylobacter jejuni* and *C. coli.* Transmission to humans is most often associated with the handling and consumption of poultry meat, but can also occur through other pathways, such as handling and consumption of contaminated water [5]. The main symptom of campylobacteriosis is mild or self-limiting gastroenteritis, but infection can also lead to immune-mediated diseases such as Guillain–Barré syndrome and reactive arthritis [1,4,6,7]. It was estimated that in 2010, *Campylobacter* spp. caused the highest number of laboratory-confirmed food-borne bacterial infections worldwide (96 million, 95% UI: 51–177) [3]. In 2010, food-borne campylobacteriosis was estimated to cause 9 DALYs per 100,000 population (95% UI: 6–13) in the WHO European Region [3].

Listeriosis is caused by the Gram-positive bacterium *Listeria monocytogenes* that, in contrast to many other food-borne pathogens, can grow at refrigeration temperatures [8]. This ability to persist and multiply in the food storage environment makes *L. monocytogenes* particularly difficult to control [8]. *L. monocytogenes* infections in healthy individuals may cause febrile gastroenteritis that is usually mild and self-limiting, but in patients with impaired immunity, it can lead to severe disease including septicaemia, meningitis or encephalitis with sequelae or death [9]. Infection during pregnancy may result in spontaneous abortions or stillbirths [10]. In 2010, listeriosis was estimated to cause two DALYs per 100,000 population (95% UI: 1–2) in the WHO European Region [3,11].

The DALY metric quantifies the burden of a disease as the number of healthy years of life lost to morbidity and mortality, and is an internationally recognised summary measure of population health. It facilitates comparing the relative impact of diseases and risk factors over time [12,13]. DALYs have been used to estimate the burden of non-communicable diseases or injury in Belgium [14-16], but they have not been used to estimate the burden of communicable diseases even though its applicability to such has been demonstrated for other European countries [17-19]. To date, neither the future global nor future Belgian burden of food-borne diseases has been predicted despite a changing, ageing population, and life expectancy possibly influencing the burden of the bacterial food-borne diseases in the coming years. The use of time series analyses, which is mainly used in economics, may be relevant for studying trends and future trends of food-borne diseases.

This study tries to address the aforementioned gaps by providing estimates of the current and future numbers of salmonellosis, campylobacteriosis and listeriosis cases, and the resulting DALYs, in Belgium from 2012 to 2020 using time series analyses. This study will generate valuable information for decision makers and researchers, provide an explanation of the development of suitable time series models for forecasting cases of food-borne infections and may be a starting point for other burden of food-borne diseases studies in Belgium.

## Methods

### Data

The Belgian Scientific Institute of Public Health (WIV-ISP) collects data on laboratory-confirmed salmonellosis, campylobacteriosis and listeriosis cases in Belgium. The National Reference Centre for *Salmonella* spp. and *Listeria* spp. of the WIV-ISP receives strains from Belgian laboratories in order to type them or define their antimicrobial resistance profile [20]. Positive results of stool cultures for *Campylobacter* spp. are collected by laboratories participating in the Belgian Sentinel Network of Laboratories (SNL) that was created in 1983 to monitor trends in infectious diseases. About 60% of Belgian laboratories participate in this sentinel system [21]. The number of cases per month were available from January 2001 to December 2012 for salmonellosis, from January 1993 to December 2013 for campylobacteriosis and from January 2011 to December 2013 for listeriosis. We included all reported cases of campylobacteriosis and salmonellosis in Belgium without distinguishing the origin of the infection, i.e. not all *Salmonella* and *Campylobacter* infections will have been caused by the consumption of contaminated food. Hald et al. estimate that for the EUR-A subregion, which includes Belgium, the proportion of salmonellosis and campylobacteriosis cases attributable to food was 76% (95% UI: 47–94%) and 76% (95% UI: 44–93) in 2010, respectively [22].

### Salmonellosis model development

The salmonellosis time series consisted of the monthly number of cases from January 2001 to December 2012. Visual inspection of the salmonellosis time series showed that there was a downward trend after 2006 and strong seasonality. Because both the Shapiro and Jarque-Bera tests rejected the normality of the salmonellosis time series distribution (p < 0.001), and because autocorrelation and partial autocorrelation plots (supplementary material [23]) indicated that the data were highly autocorrelated and seasonal, we needed a nonlinear, non-Gaussian time series model.

Collard et al. [24] previously reported a dramatic drop in salmonellosis cases in Belgium after 2005. They noted that in 2003, Belgium adopted changes in the breeder-flock poultry monitoring and control programme, with a possible influence on *Salmonella* transmission and control. Furthermore, since 2003, a poultry vaccination programme has been in place [24]. However, the effects of these policy changes could not be dated exactly, meaning that the time series was not easy to segment into forecastable sub-series and that the data exhibited possible non-stationarity or unstable behaviour [25], therefore requiring the need for a change-point model. We selected the Bai and Perron [26,27] change-point model for autoregressive time series models allowing for structural changes in the parameters. The time series’ partial autocorrelation function (PACF) and autocorrelation function (ACF) related to the Bai and Perron model resulted in stationarity (supplementary material [23]), suggesting that the change-point model could explain the intense changes in the dynamics, levels and trends in the data. The model identified the number and location (in time) of the breakpoints. Since adding more breakpoints will always result in lower errors in a regression context, a Bayesian information criterion (BIC) was used for final model selection to ensure the selection of a model with an optimal set of breakpoints [26]. The choice of the number and location of the lagged values used in the model was made such that the residuals of the final regression were white noise or serially uncorrelated.

The final model with serially uncorrelated residuals for the series $S_{t}$ had the form:

$$S_{t}=c+\;\phi_{1j}S_{t-1}+\phi_{2j}S_{t-2}+\phi_{12j}S_{t-12}+\frac{\beta_{j}t}{T}+\epsilon_{t}$$

where the sub-samples for the *m* breakpoints are defined by the indices at time *t*,

*t* = *t_j-1_* + 1, …, *t_j_*, *j* = 1, …, *m* + 1

This regression had m sets of regression coefficients for the m+1 subsets of the data. The lagged *S_t_* terms captured the first and second order and seasonal autoregressive effects. The *c* and $\phi_{1j}$, , $\phi_{12j}$ are the intercept and autoregressive terms for the effects of the lagged values at times *t−1, t−2*, and *t−12*. These autoregressive terms captured the lagged effects of the previous 2 months and the seasonal correlation across the same month the previous year. The normalised trend term $\beta_{j}t$ was allowed to vary across the regimes allowing for determination of whether the trend increased or decreased over the data.

### Campylobacteriosis model development

**Figure 1:**
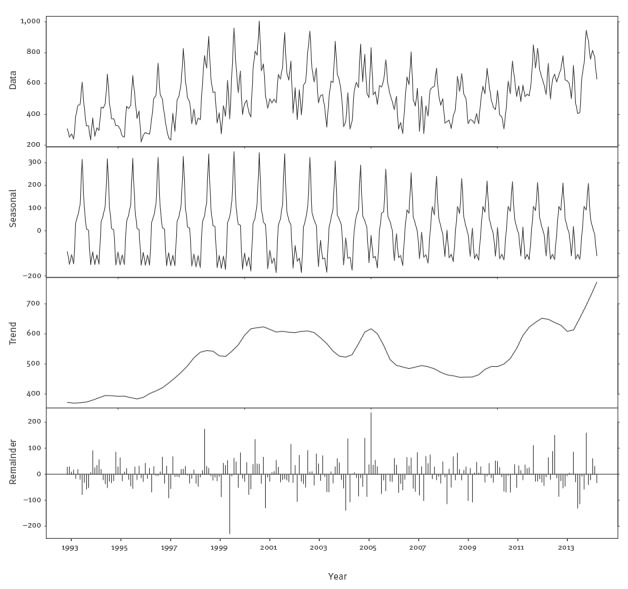
Seasonal-trend-loess (STL) decomposition of monthly Belgian campylobacteriosis data, Belgium, 1993–2013

The campylobacteriosis time series consisted of the monthly number of cases from December 1993 to December 2013. Visual inspection of the campylobacteriosis time series showed a time-varying behaviour and a strong seasonality. As for salmonellosis, we first investigated the distributional properties of the data. The PACF and ACF for the campylobacteriosis time series indicated strong seasonality, as well as low order autocorrelation since there were significant spikes at the low order lags (1–4 in the PACF plot) (supplementary material [23]). A time-varying behaviour was indicated by shifts in the trend over time (supplementary material [23]), suggesting that a dynamic linear model (DLM) was appropriate. While the autoregressive integrated moving average (ARIMA) models may be appropriate for fitting these data, such models would forecast poorly since they assume constant parameters. Further, for the data at hand, ARIMA models resulted in coefficients indicating borderline non-stationarity. The trend in these data had a stochastic drift and varied locally, increasing at an earlier stage in the sample and at the end, but staying relatively constant in between. The DLM dealt with potential issues, estimated the time-varying trend and recognised that there were parameter stability issues around the seasonal component of the data. The DLM was made up of separate components for the drift, trend and cyclical components of the time series. With a DLM, these separate components were filtered out of the data resulting in a forecasting model with both fixed and time-varying parameters. As Figure 1 shows, the seasonal component of the data was not constant over time, and the seasonal variation has been lower since 2007. Second, there is a strong upward trend in the data as the trend ranges from 4,000 to nearly 8,000 cases per year and fluctuates over time, further supporting the choice of a time-varying parameter model like a DLM.

The DLM contains three components: a stochastic linear trend, a time-varying set of deterministic dummy variables for the seasonal component and a first order autoregression. The linear trend allowed for capturing the changing trend seen in Figure 1. The seasonal terms captured the time-varying seasonality in Figure 1. Finally, the autoregression captured the serial correlation reported in the supplementary material [23]. The resulting model was fitted via maximum likelihood methods.

### Listeriosis model development

The listeriosis time series counted monthly cases from January 2011 to December 2013. Visual inspection of the listeriosis time series showed a stochastic distribution of the cases. This was a relatively short time series so a comparison of both a dynamic or time series model and a static prediction were in order. The short available data time span of 36 months did not enable us to obtain reliable estimates of seasonal or other dynamic patterns, and only allowed us to reasonably forecast for one year. While it would appear that there might be some seasonality, the testing for serial correlation in the series via ACFs (supplementary material [23]) showed no significant serial correlation in the time series.

The lack of significance for serial correlation via ACF tests did not mean that there was no serial correlation in the count series. To better investigate the serial correlation aspects, a series of Poisson autoregressive (PAR(p)) models were fitted to the data. The main choice in employing the model is the order of the lag, the integer value of p. In this analysis, values of p from one to four were tested. The best fitting model was chosen based on the statistical significance of the estimated autoregressive coefficients and was a PAR(2) model. Predictions from this model were made by simulation using direct forecasting via the mean prediction function from Brandt and Williams [28].

A simulation of 1,000 forecast paths was employed for the three developed models, one for each disease, to generate mean forecasts and to compute a confidence region for them.

### DALY calculations

The burden of salmonellosis, campylobacteriosis and listeriosis was evaluated in terms of DALYs. DALYs were calculated according to the standard formulas [29], without age weighting and time discounting. Standard expected years of life lost were based on the Global Burden of Disease (GBD) 2010 model life table [30], while Belgian life tables were used for estimating lifelong durations of sequelae [31]. We calculated DALYs in 2012 based on observed number of cases, and DALYs in 2014 and 2020 based on the number of cases estimated by the three models developed above. The incidence calculations were based on a Belgian population of 11,161,642 inhabitants in 2012, 11,226,322 inhabitants in 2014 and a prediction of 11,364,047 inhabitants in 2020 based on United Nations estimations assuming a medium fertility rate [32]. We did not correct for comorbidity, but we did correct for under-reporting of salmonellosis and campylobacteriosis cases in Belgium using the under-reporting factors (URF) developed for Belgium, 3.5 (95% UI: 0.3–11.3) for salmonellosis and 10.5 (95% UI: 3.2–26.5) for campylobacteriosis [33]. We did not correct the listeriosis time series for under-reporting as the severity of the cases was assumed to imply perfect reporting.

The DALY calculations were conducted in R version 3.1.1 using the FERG package [34]. Uncertainty was propagated using 1,000 Monte Carlo simulations and results were presented as the mean and 95% UI of the resulting uncertainty distributions.

### Outcome trees

**Figure 2:**
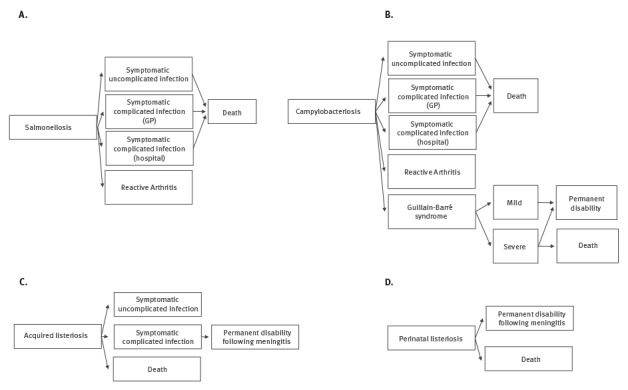
Outcome trees used for disability-adjusted life year calculations of campylobacteriosis, salmonellosis and listeriosis (acquired and perinatal), Belgium

DALY: disability-adjusted life year; GBS: Guillain–Barré syndrome: GP: general practitioner.

To estimate the full burden caused by a pathogen, all health outcomes of the infection and their possible transitions were considered by using an outcome tree. We based our outcome trees and transition probabilities on those suggested by the European Centre for Disease Prevention and Control (ECDC) [35] (Figure 2) for salmonellosis, campylobacteriosis and listeriosis because these probabilities were not available specifically for Belgian population. The proportion of perinatal listeriosis cases, defined as materno-fetal cases including pregnancy-associated cases and cases in newborns during the first month of life, was taken from Maertens de Noordhout et al. [11]. A materno-fetal infection with *L. monocytogenes* was counted as one case in the time series. We adopted durations and disability weights (DWs), the latter based on a scale from zero to one where 0 is a health state equivalent to full health and 1 is a health state is equivalent to death, as proposed by ECDC [35]. For salmonellosis and campylobacteriosis, we used a DW of 0.073 (95% confidence interval (CI): 0.061–0.092) for ‘symptomatic uncomplicated infections’, 0.149 (95% CI: 0.120–0.182) for ‘symptomatic complicated infections (general practitioner (GP))’, 0.239 (95% CI: 0.202–0.285) for ‘symptomatic complicated infections (hospital)’ [36], 0.344 (95% CI: 0.300–0.390) for ‘reactive arthritis’ [37], 0.053 (95% CI: 0.042–0.064) for ‘Guillain–Barré syndrome, mild’, 0.520 (95% CI: 0.465–0.581) for ‘Guillain–Barré syndrome, severe’ and 0.421 (95% CI: 0.377–0.477) for ‘permanent disability following Guillain–Barré syndrome’ [38]. For listeriosis we used a DW of 0.149 (95% CI: 0.120–0.182) for ‘symptomatic uncomplicated infection’, 0.655 (95% CI: 0.579–0.727) for ‘symptomatic complicated infection’ and a 95% CI of 0.011–0.421 for ‘permanent disability due to meningitis’ [39].

Outcome trees were designed according to data availability and those suggested by the European Centre for Disease Prevention and Control (ECDC) [35].

## Results

### Salmonellosis

#### Model performance and incidence forecast

Fitting up to a five breakpoint model (m=5), the optimal BIC was produced for a two breaks model. The estimated breaks were in December 2003 and in November 2005, consistent with the results reported in Collard et al. [24]. Table 1 shows the estimated parameters over each of the periods.

**Table 1:** Bai and Perron change-point regression estimates for salmonellosis cases, Belgium, 2001–2012

Coefficient	Jan 2001– Nov 2003	Standard error	p value	Dec 2003– Oct 2005	Standard error	p value	Nov 2005– Dec 2012	Standard error	p value
**Intercept**	144.35	51.94	0.006	626.36	114.70	< 0.001	44.56	50.39	0.378
*Φ_1j_*	1.20	0.10	< 0.001	0.06	0.19	0.759	0.67	0.14	< 0.001
*Φ_2j_*	−0.83	0.08	< 0.001	0.05	0.13	0.717	−0.24	0.12	0.049
*Φ_12j_*	0.32	0.08	< 0.001	0.54	0.08	< 0.001	0.36	0.09	< 0.001
*β_j_*	2106.07	456.20	< 0.001	−2261.68	375.70	< 0.001	14.54	44.90	0.747
**Standard error**	73.99								
**R^2^**	0.99								

We observed a strong autoregressive model with seasonality, there were relatively more salmonellosis cases from June to August than in other months, and a statistically significant positive trend (p < 0.001) during the period January 2001 to November 2003.

The dynamics of the second period, from December 2003 to October 2005, was different compared with the dynamics of the first period. The first two autoregressive lags were not significant, but there was a strong seasonal component (p < 0.001). The trend became negative in this period, meaning that the number of cases decreased, reflecting the poultry vaccination. During this second period, the estimated trend coefficient was negative and larger in absolute value than the upward trend of the first period.

In the last period from November 2005 to December 2013, a new equilibrium was reached after the drop brought by the changes in vaccination and hygiene policies. There was no statistically significant intercept or trend. Instead, the autoregressive dynamics dominated the process. The seasonality became much weaker in the third segment of the change-point model and explained why the seasonality is much reduced in the prediction. It has a much stronger AR(1) and AR(2) component that oscillates in the third period and smooths out to a long-run equilibrium.

**Figure 3:**
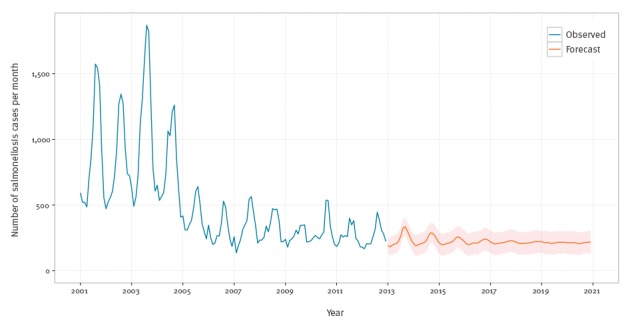
Monthly observed and forecasted salmonellosis cases, Belgium, 2001–2020

Figure 3 presents the in-sample fit as well as a 96 month (8 year) forecast of salmonellosis cases through to the end of 2020. The time series is well fitted by this model (see supplementary material [23]), and the forecast shows a continued decline to the long-run means after 2005. Figure 3 also includes simulated one standard error bands. The 2012 average number of confirmed cases per month was 264 (standard deviation (SD): 86) and the final equilibrium prediction for 2020 was 212 cases (SD: 87) per month.

Blue lines are observed. Orange lines are forecasts. Orange areas are simulated one standard deviation error bands.

**Figure 4:**
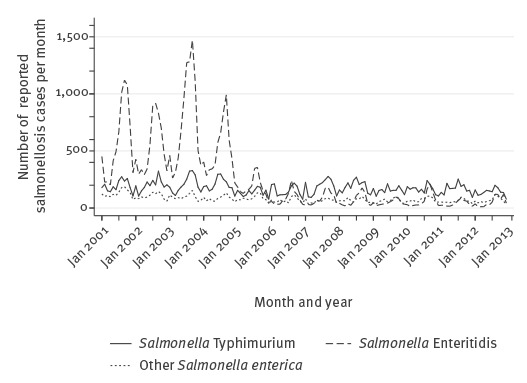
Monthly confirmed salmonellosis cases by species, Belgium, 2001–2012

It is of note that in the aggregated data used in the analysis, the trend is dominated by *S. enterica* subspecies *enterica* serovar Enteritidis, with numbers decreasing to very low levels, whereas the number of cases caused by *S. enterica* subspecies *enterica* serovar Typhimurium and other *S. enterica* remains constant across the series (Figure 4).

#### DALYs estimates

We estimated that in 2012, salmonellosis caused 102 DALYs (95% UI: 8–376) or 0.9 DALYs per 100,000 population (95% UI: 0.07–3). Based on our forecast model, the burden in 2020 would drop to 82 DALYs (95% UI: 6–310) or 0.7 DALYs per 100,000 population (95% UI: 0.05–3) (Table 2).

**Table 2:** Current cases, future cases and disability-adjusted life years for salmonellosis, campylobacteriosis and listeriosis, Belgium, 2012–2020

Pathogen	2012							2020 (salmonellosis and campylobacteriosis) and 2014 (listeriosis)							
	Reported cases (n)	Years lived with disability (net values)		Years of life lost (net values)		Disability-adjusted life years (net values)		Predicted cases (n)	SD	Years lived with disability (net values)		Years of life lost (net values)		Disability-adjusted life years (net values)	
		Mean	95% UI	Mean	95% UI	Mean	95% UI			Mean	95% UI	Mean	95% UI	Mean	95% UI
**Non-typhoidal *Salmonella enterica^a^***	NA	44	4–162	58	2–238	102	8–376	NA	NA	35	3–132	47	2–182	82	6–310
***Campylobacter* spp.^a^**	NA	379	67–1,295	640	44–2,023	1,019	137–3,181	NA	NA	651	89–2,381	1,085	59–3,555	1,736	178–5,874
***Listeria monocytogenes* (acquired)**	64	14	2–29	193	185–200	208	192–226	74	6	17	2–36	235	191–282	252	200–307
***Listeria monocytogenes* (perinatal)^b^**		56	7–115	213	185–248	269	208–347			54	6–107	204	188–223	258	204–322
**Non-typhoidal *Salmonella enterica^c^***	3,170	13	7–24	17	2–33	30	10–54	2,442	440	10	5–20	14	1–29	24	7–46
***Campylobacter* spp.^c^**	7,598	37	14–101	62	7–121	98	23–203	12,909	3,612	63	16–205	104	8–252	167	28–394

NA: not applicable; SD: standard deviation; UI: uncertainty interval.

^a ^With correction for under-reporting of 3.50 (95% UI: 0.34–11.30) for salmonellosis and 11.50 (95% UI: 3.20–26.50) for campylobacteriosis.

^b^ Perinatal listeriosis cases were defined as materno-fetal cases including pregnancy-associated cases and cases in newborns during the first month of life. Materno-fetal infection with *L. monocytogenes* was counted as one case in the burden of disease estimates.

^c^ Without correction for under-reporting.

### Campylobacteriosis

#### Model performance and incidence forecast

**Figure 5:**
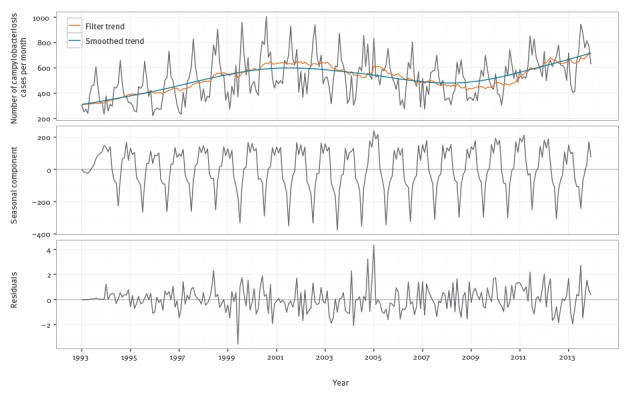
Fitted dynamic linear model (DLM) and decomposition for the Belgian campylobacteriosi*s* series, Belgium, 1993–2013

Figure 5 presents the components of the selected DLM model, i.e. trend and seasonal components, being computed recursively with one-step ahead updates using the Kalman filter. The top panel of the figure shows the data and the filtered and smoothed trend estimates. The middle panel gives the time-varying seasonal component. The general seasonal pattern is the same, but the magnitudes of the seasonality changed over time. The last panel shows the residuals. Ljung–Box tests for serial correlation failed to reject the null hypothesis of serial correlation at multiple lag lengths (lag 1, p = 0.53; lag 2, p = 0.55; lag 12, p = 0.31). Since this model had white noise residuals and captured the main trend, cyclical features and seasonality of the data, it was considered the best candidate for forecasting the number of campylobacteriosis cases in Belgium.

**Figure 6:**
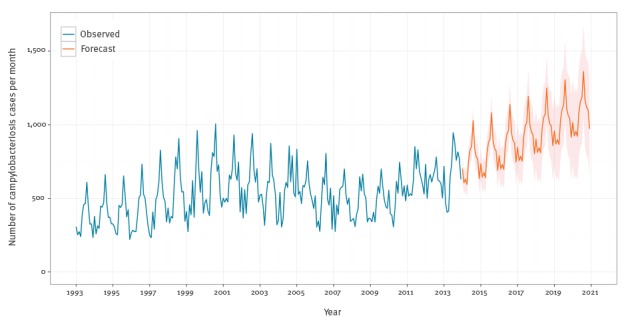
Monthly observed and forecasted campylobacteriosis cases, Belgium, 1993–2020

Figure 6 shows the campylobacteriosis forecasts through 2020. The upward trend was extrapolated from the earlier analysis in Figure 1. The seasonal component is seen in the middle panel of Figure 5.

Blue lines are observed. Orange lines are forecasts. Orange areas are simulated one standard deviation error bands.

The average monthly number of campylobacteriosis confirmed cases was 633 (SD: 81) in 2012, with the predictions showing an upward trend until 2020 to an average of 1,081 (SD: 311) cases per month. The predictions showed that the campylobacteriosis cases will exceed their previous peak of 1,005 cases in August 2000 in May 2019 (Figure 6).

#### DALYs estimates

We estimated that campylobacteriosis caused 1,019 DALYs (95% UI: 137–3,181) or 9 DALYs per 100,000 population (95% UI: 1–29) in 2012, which would increase to 1,736 DALYs (95% UI: 178–5,874) or 15 DALYs per 100,000 population (95% UI: 2–52) in 2020. In 2012, 63% of the burden was caused by years of life lost (Table 2).

### Listeriosis

#### Model performance and incidence forecast

Table 3 shows the estimated coefficients and fit for this model. The Wald test for comparison with a static Poisson regression had a value of 15.46 with a p value less than 0.001, indicating that this model is preferred over a standard Poisson regression model. The estimated autoregressive parameters for this model, ρ1 and ρ2 were negative, meaning there were fewer predicted listeriosis cases after they were observed.

**Table 3:** PAR(2) estimates for listeriosis time series, Belgium, 2011–2013

Coefficient	Estimate	Standard error	Z-score
**ρ_1_**	−0.28	0.13	−2.17
**ρ_2_**	−0.24	0.15	−1.67
**Intercept**	1.83	0.06	32.11

**Figure 7:**
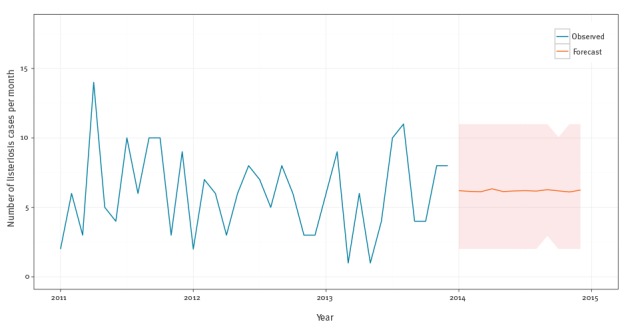
Monthly observed and forecasted listeriosis cases, Belgium, 2011–2014

The mean forecast is 74 cases (SD: 6) in 2014 or six new additional cases per month (SD: 2.6) in 2014 (Figure 7).

Blue lines are observed. Orange lines are forecasts. Orange areas are simulated one standard deviation error bands.

#### DALYs estimates

Acquired listeriosis was estimated to have caused 208 DALYs (95% UI: 192–226) or two DALYs per 100,000 population (95% UI: 1–2) in 2012 and was predicted to cause 252 DALYs (95% UI: 200–307) or two DALYs per 100,000 population (95% UI: 2–3) in 2014. Perinatal listeriosis was estimated to have caused 269 DALYs (95% UI: 208–347) or two DALYs per 100,000 population (95% UI: 2–3) in 2012 and was predicted to cause 258 DALYs (95% UI: 204–322) or two DALYs per 100,000 population (95% UI: 2–3) in 2014. Most of the burden is caused by mortality (Table 2).

## Discussion

Using time series analysis, we predicted that the burden of salmonellosis will remain stable between 2012 and 2020, that the burden of listeriosis remained stable between 2012 and 2014, but that the burden of campylobacteriosis would increase by a factor of almost two by 2020.

Our study also showed that a time series analysis is an appropriate methodology to help reveal or clarify trends of food-borne illnesses, to forecast future cases and to test the impact of interventions on the burden of food-borne diseases. Time series models could complement the public health surveillance of food-borne diseases [40,41], and could also be used to identify irregularities in disease incidence [42,43]. Model application can also result in more efficient and cost-effective control strategies [44].

To our knowledge, this study is the first that used and developed segmented regressions, i.e. a Bai and Perron model [26], to predict food-borne diseases and that predicted salmonellosis cases and DALYs. Others have already predicted future DALYs of non-food-borne diseases using a demographic dynamics model [45], future years lived with disabilities (YLDs) caused by *Salmonella* spp. infection using future temperature change prediction [46] and examined seasonal and secular trends of enteric diseases using spatio-temporal analysis [47]. We estimated that salmonellosis caused 0.9 DALYs per 100,000 population (95% UI: 0.07–3) in 2012 in Belgium. This is roughly consistent with the estimations of salmonellosis resulting in 3 DALYs per 100,000 population (95% UI: 3–5) in 2010 in the WHO European Region [3] but less than the estimates of 7 DALYs per 100,000 population (95% UI: 5–10) in 2012 in Denmark [19], 23 DALYs per 100,000 population (95% UI: 18–30) in 2007 in Germany [48], and 8 DALYs per 100,000 population (95% UI: 4–17) in 2011 in the Netherlands [49]. These differences are caused by differences in the outcome trees used. Indeed, Irritable bowel syndrome (IBS) and Inflammatory bowel disease (IBD) are not included in the new outcome trees developed by ECDC because it concluded that there was not enough evidence that salmonellosis resulted in IBS and IBD [35].

When we included IBS in our outcome tree, the DALYs caused by salmonellosis in Belgium in 2012 increased by 107% (supplementary material [23]). The differences can be further explained by the fact we adjusted the salmonellosis incidence for under-reporting using an URF developed by Havelaar et al. [33]. This allowed us to compare the burden of salmonellosis with the burden caused by other diseases, such as listeriosis. This URF developed by Havelaar et al. for salmonellosis is particularly low for Belgium (3.5); by comparison, the URFs used in the Danish (7.2) [19], German (8.7) [48] and the Dutch (18) [49] studies were higher. Using URFs developed for the Netherlands or Germany in the scenario analysis, the DALYs caused by salmonellosis increased by 653% or 203%, respectively (supplementary material [23]). If we both included IBS as a sequela of salmonellosis and used the URF developed for the Netherlands, we observed an increase in DALYs of 1456% (supplementary material [23]). The case–fatality rate used for DALYs estimations may also be a reason for the observed differences in DALYs. For instance, Van Lier et al. used an average case–fatality rate of 0.10% in the Netherlands [49], which is higher than what we used (min 0.05%– max 0.10%). Based on the above, it is clear that different methodologies used for burden of salmonellosis estimates make comparison between countries difficult. There is a need to develop more precise URFs for the Belgian population because the uncertainty in the URFs of salmonellosis remains high, and given the one-to-one relationship with the DALY estimate, very influential.

This is the first time that DLM models were used to predict food-borne diseases cases and DALYs. Weizent et al. also concluded that decomposition models, like DLM, are more appropriate models for forecasting campylobacteriosis risk in the United States than regression or Box-Jenkins ARIMA models [50]. Nobre et al. applied DLM models to malaria and hepatitis A data, and concluded that DLM models were adequate tools for use in epidemiological surveillance [51]. Using a DLM, we estimated that campylobacteriosis caused nine DALYs per 100,000 population (95% UI: 1–29) in 2012. This is equal to the WHO estimates for the WHO European Region (9/100,000 population; 95% UI: 6–13) and consistent with the estimates for the Netherlands in 2011 (2/100,000 population; 95% UI: 8–41) [49] and for Denmark in 2012 (28/100,000; 95% UI: 25–33) [19].

We developed a Poisson autoregressive model for listeriosis cases given the short time series. This is the first time severe listeriosis burden has been estimated in Belgium. We estimated that acquired listeriosis caused four DALYs per 100,000 population (95% UI: 3–4) in both 2012 and 2014. Perinatal listeriosis was estimated to cause two DALYs per 100,000 population (95% UI: 2–3) in both 2012 and in 2014. These results are in line with those of Maertens de Noordhout et al. who estimated that listeriosis caused thee DALYs per 100,000 population (95% UI: 2–3) in 2010 in WHO European sub-region A, which includes Belgium [11]. In 2014, the Belgian Scientific Institute of Public Health reported 83 cases of listeriosis which falls within one SD from what we predicted for the same year using the Poisson model (74 cases, SD: 6.1) [52]. However, our listeriosis estimates could have been underestimated because we did not correct for under-reporting as the severity of the cases was assumed to imply perfect reporting. Some listeriosis cases could be uncomplicated, especially in healthy people, and thus not be reported. Correcting the reported cases of listeriosis in Belgium in 2012 by the URF developed by Thomas et al. for Canada [53] increased the DALYs caused by acquired and perinatal listeriosis by 10% (supplementary material [23]).

Although this study estimated, for the first time, the DALYs linked to salmonellosis and campylobacteriosis in 2012 and 2020, and to listeriosis in 2012 and 2014 in Belgium, it also had several limitations. First, a major limitation of prediction models is that they implicitly assume that the environment and context will stay stable. Changes in knowledge about safe food handling, physician testing practices, new public health or animal health interventions or future outbreaks could all violate this assumption, but are impossible to predict [54]. Second, we did not include food attribution percentages in the burden estimates, which means that some campylobacteriosis, salmonellosis and listeriosis cases may have been attributed to other sources of contamination than the consumption of contaminated food. Third, potential clustering of salmonellosis, which is more common compared with the other two diseases, was not taken into account in the times series analysis. Indeed, in 2012, the European Food Safety Authority reported 1,533 *Salmonella* spp. outbreaks in Europe, which is higher than the reported number of outbreaks caused by *Campylobacter* spp. (n = 501) or by *L. monocytogenes* (n = 5) [55]. The presence of clustering will, in general, not influence the estimates, but it may have an influence on the UI. Fourth, while we used projected life expectancies for sequelae with a lifelong duration, we used the same transition probabilities and age distribution of cases for the forecasted 2020 and estimated 2012 DALY calculations. Of course, all parameters would be influenced by many factors, including new treatments, improved management of food-borne diseases, ageing of the population [45] or climate changes [56].

The precision of our time series models could be further improved by including relevant covariates; for instance, data source, diagnostic method, sex, age, prevalence in animals, prevalence in food, or consumption of proton-pump inhibitors that can increase the risk of camplylobacteriosis, salmonellosis or listeriosis [57,58] could potentially be included in the model. If such data are available and included, it would reduce the uncertainty of the predictions and produce more informed extrapolations. This may in turn increase the precision of our estimations, but would require a re-evaluation of the selected model, especially for listeriosis as little data were available for this at the time of the study.

## Conclusion

Assuming a constant environment, e.g. no change in policy and control of salmonellosis, campylobacteriosis and listeriosis, the incidence of salmonellosis and listeriosis is predicted to remain stable in Belgium, while the incidence of campylobacteriosis may almost double until 2020. Efforts to control cases of salmonellosis and listeriosis in Belgium must be maintained in the future whereas new actions are urgently needed to understand and reduce the risk of food being contaminated with *Campylobacter* spp. This study is also a starting point for other studies that wish to project the future burden of disease of other food-borne pathogens, and for the Belgian national burden of disease study that was launched at the end of 2016.
